# Global Metabolomics Reveals the Metabolic Dysfunction in Ox-LDL Induced Macrophage-Derived Foam Cells

**DOI:** 10.3389/fphar.2017.00586

**Published:** 2017-08-31

**Authors:** Wenjuan Xu, Ziyi Wei, Jiaojiao Dong, Feipeng Duan, Kuikui Chen, Chang Chen, Jie Liu, Xiaowei Yang, Lianming Chen, Hongbin Xiao, An Liu

**Affiliations:** ^1^Institute of Chinese Materia Medica, China Academy of Chinese Medical Sciences Beijing, China; ^2^School of Chinese Materia Medica, Beijing University of Chinese Medicine Beijing, China

**Keywords:** metabolomics, macrophage-derived foam cells, oxidized-LDL, atherosclerosis, anandamide over-accumulation

## Abstract

Atherosclerosis (AS) is a chronic disorder of large arteries that is a major risk factors of high morbidity and mortality. Oxidative modification LDL is one of the important contributors to atherogenesis. Macrophages take up ox-LDL and convert into foam cells, which is the hallmark of AS. To advance the understanding of the metabolic perturbation involved in ox-LDL induced macrophage-derived foam cells and discover the potential biomarkers of early AS, a global metabolomics approach was applied based on UHPLC-QTOF/MS. Multivariate statistical analyses identified five metabolites (25-azacholesterol, anandamide, glycocholate, oleoyl ethanolamide, and 3-oxo-4, 6-choladienoate) for distinguishing foamy macrophages from controls. Among the six main metabolic pathways, the unsaturated fatty acid, especially arachidonic acid metabolism, contributed importantly to early AS. A new biomarker, anandamide (AEA), whose synthesis and metabolism in macrophages are disturbed by overloaded ox-LDL, results in metabolic obstruction. This study is the first to investigate the metabolic disturbance in macrophage-derived foam cells induced by ox-LDL and screen potential biomarkers and metabolic pathways associated with early AS. Our findings provide a new insight in the underlying pathophysiological mechanisms and also help to identify novel targets for the intervention of AS.

## Introduction

Atherosclerosis (AS) is a chronic disorder of large arteries which underpins the development of important vascular diseases, such as coronary and cerebrovascular diseases (Rocha and Libby, 2009; Furie and Mitchell, 2012). Now, there is a consensus that inflammation and elevated oxidative stress are the major state of AS (Stocker and Keaney, 2004; Feng et al., 2011). Plasma LDL is transported into the arterial wall and then subjected to oxidative modifications, which is one of the first steps of AS. Oxidized low density lipoprotein (ox-LDL) is a potent inflammatory molecule inducer and is considered to be the typical atherogenic form of LDL (Catapano et al., 2000; Steinberg, 2009). Thus, lowering plasma LDL levels and inhibiting LDL oxidation have become a focus in the prevention and therapeutic intervention of AS. More recently, increasing evidence has demonstrated that ox-LDL promoted monocytes to differentiate into macrophages and was taken up by macrophages via scavenger receptors (SR-A, CD68, CD36), resulting in lipid accumulation thus foam cell formation (Han et al., 1997; Parthasarathy et al., 2001). Numerous studies have been conducted to investigate the mechanisms of ox-LDL-induced foamy macrophage formation and the potential link between cholesterol accumulation and inflammation. However, most of previous studies mainly focused on the effect of various types of scavenger receptors, cytokines and chemokines (Xia et al., 2013; Li X.Y. et al., 2016). But what happens within macrophages? How does ox-LDL affect the metabolic alteration in foamy macrophages? AS is a typical metabolic disorder and its metabolic perturbations will inevitably be reflected in endogenous metabolites; however, the metabolic characteristics of ox-LDL-induced foamy macrophages remain poorly understood. It is necessary and of great practical significance to elucidate the relevance between ox-LDL exposure and metabolic perturbations, and identify a pool of potential biomarkers of macrophage-derived foam cells.

Metabolomics as an attractive tool has been widely applied in revealing the global metabolic networks and cross-regulations (Li Z. et al., 2016; Zhang et al., 2016). Hence, exploration of the feedback effect of terminal endogenous metabolites on ox-LDL overload is expected to be a practicable way to ascertain new therapeutic targets for AS and its complications. Nevertheless, there is still a lack of metabolomics data on ox-LDL-induced foamy macrophages. Therefore, in this study, a cell model of ox-LDL induced foamy macrophages was developed and analyzed by global metabolomics using ultra-performance liquid chromatography coupled with quadrupole time of flight mass spectrometry. Meanwhile, pathway analysis was employed to visualize the metabolic disturbances involved in foamy macrophages systematically. Our study provides a more global view of the metabolic perturbations in macrophage-derived foam cells, which may guide us toward a better understanding of the pathogenesis underlying macrophages response to lipid deposition and AS formation.

## Materials and Methods

### Chemicals

Fetal bovine serum and High-glucose Dulbecco’s Modified Eagle medium (DMEM) used in cell culture were purchased from HyClone. Phosphate buffer saline (PBS), trypsin/EDTA solution and dimethylsulfoxide (DMSO) were obtained from Solarbio (Beijing, China); 3-(4, 5-Dimethylthiazol-2-yl)-2, 5-diphenyltetrazolium bromide (MTT) was purchased from Amresco (United States). Protein quantitative test kit was obtained from Applygen (Beijing, China). Ox-LDL and oil red O were both bought from Yiyuan Biotechnologies (Guangzhou, China). Total cholesterol (TC) and free cholesterol (FC) assay kit were purchased from Applygen (Beijing, China). Acetonitrile and methanol for HPLC analysis were purchased from Fisher Chemical (United States); water used for UPLC was prepared from Milli-Q ultra-pure water system (Millipore, Billerica, MA, United States); HPLC grade formic acid was obtained from Fluka (United States).

### Cell Culture and Treatment

Macrophages, Raw 264.7 were provided by National Infrastructure of Cell Line Resource (Beijing, China), and cultured with high-glucose DMEM supplemented with 10% fetal bovine serum and maintained in a 37°C humidified atmosphere under 5% CO_2_ until subconfluent. Cells were seeded into 6-well culture plates at a density of 1 × 10^6^ cells/well. After treating with ox-LDL (50, 100, and 150 μg/mL) for 24 h, cells were subjected to oil-red O staining and cholesterol determination.

### Oil-Red O Staining

Briefly, Raw 264.7 cells were loaded with ox-LDL for 24 h to observe lipid droplets. Macrophages were fixed for 20 min using 4% paraformaldehyde, washed with PBS for three times, and then treated with oil-red O (dissolved in 60% isopropanol, 0.5% w/v) for 30 min. Cells were harvested and prepared routinely for observation of lipid droplets in ox-LDL derived foam cells using an Olympus IX71 microscope (Olympus).

### Cellular Cholesterol Analysis

Total cholesterol and FC levels were measured by a cholesterol assay kit. The cells were harvested and washed with PBS for three times. Reference cholesterol (36-625 μ mol/L) was used to draw a standard curve. Cholesteryl ester (CE) were obtained by subtracting the FC from the TC. CE levels were normalized to protein contents. Six independent replicates were performed for each experiment.

### Cell Viability Assay

Raw 264.7 cells with a density of 1 × 10^4^ cells/well were seeded into 96-well culture plates. Ox-LDL of different doses were added into medium to induce foam cell formation. Cells were washed with PBS after treatment for 24 h and then incubated with MTT (dissolved in PBS, 0.5 mg/mL) for 4 h. Then the medium was discarded and the formazan were dissolved in 150 μL DMSO. Absorbance value was recorded at 490 nm using Infinite 200 PRO NanoQuant (Tecan, Swiss). Six independent replicates were performed for each group.

### Intracellular Metabolite Extraction

Cell samples were harvested and washed three times with PBS. Then 400 μL methanol (75%, v/v) was successively added to each sample to break up using Omni Bead Ruptor 24 (OMNI, United States) (homogenization for 20 s, interval for 20 s, third). Then MTBE (1 mL) was added into cell samples and shaken for 1 h. Water (250 μL) was added in the mixture to induce the phase separation, standing for 10 min at room temperature and centrifuging at 14,000 × *g* for 15 min at 4°C. The two phases were separately transferred to fresh tubes and the mix supernatant (320 μL upper plus 320 μL lower fraction) was collected and dried in speed-vac, then reconstituted with 200 μL of 20% methanol. Nine independent replicates were performed for each group for metabolomics research.

### UPLC/Q-TOF MS Analysis

Intracellular metabolic profile was analyzed on an Agilent 1290 UPLC-tandem 6550 Q-TOF/MS system (Agilent, United States). In this study, chromatographic separation was carried out on Waters ACQUITY T3 column (2.1 mm × 100 mm, 1.8 μm). Mobile phase A was water and phase B was acetonitrile, both were with 0.1% formic acid. The column temperature was kept at 35°C. The gradient of mobile phase B was as follows: 0–8 min, 5–45%; 8–12 min, 45–80%; 12–16 min, 80–100%; 16–20 min, 100%. The quality control (QC) was prepared by pooling together 20 μL aliquots of all individual cell samples in order to assess repeatability and stability of sample pretreatment and analysis. The flow rate was 0.3 mL/min, and all samples were injected randomly with a 5 μL injection volume.

Mass spectrometry acquisition was performed on Agilent Q-TOF 6550, equipped with Agilent Jet Stream ESI source. The scan range was from 80 to 1700 and data acquired rate was 1 spec/sec for positive and negative ion mode. The detailed parameters were as follows: nebulizer pressure, 45 psi; sheath gas temperature, 350°C; sheath gas flow, 12 min/L; gas temperature, 250°C; gas flow, 11 min/L; capillary voltage of 4000 V (+) and 3500 V (-). The real-time internal references were used to modify precise molecular weight.

### Data Processing and Statistical Analysis

The mass spectra were extracted and analyzed via MassHunter Qualitative Analysis Software (B.04.00, Agilent). The data would be extracted and exported as a CEF file and every peak was given an identified retention time and m/z. The typical data processing steps, peak detection and alignment were performed by Mass Profiler Professional (Version 13.1, Agilent). The specific metabolites were identified by Agilent METLIN Personal Metabolite Database comparison. Peak areas were calibrated by protein content and then normalized by log transformation and pareto scaling when necessary. CV% (coefficients of variation) of variables higher than 30%, were removed from further analysis (Chan et al., 2011).

Wilcoxon Mann–Whitney test was conducted using SPSS statistics (Version 13, SPSS Inc.). The multivariate statistics (partial least squares-discriminant analysis, PLS-DA) was performed by the SIMCA-P 13.0 software (Umetrics, Umea, Sweden) (Galtier et al., 2011). Response permutation test was performed to avoid overfitting of PLS-DA model. A higher value of variable importance in the projection (VIP) indicated a higher influence of the corresponding variable. In this study, the metabolites with VIP values higher than 1.0 were regarded as significant difference. Heatmap generation was based on MetaboAnalyst^1^ (Xia et al., 2015). Correlation metabolic network construct based on metscape, a plugin of Cytoscape^2^ was performed to visualize the evolutionary profiles (Shannon et al., 2003).

## Results

**FIGURE 1:**
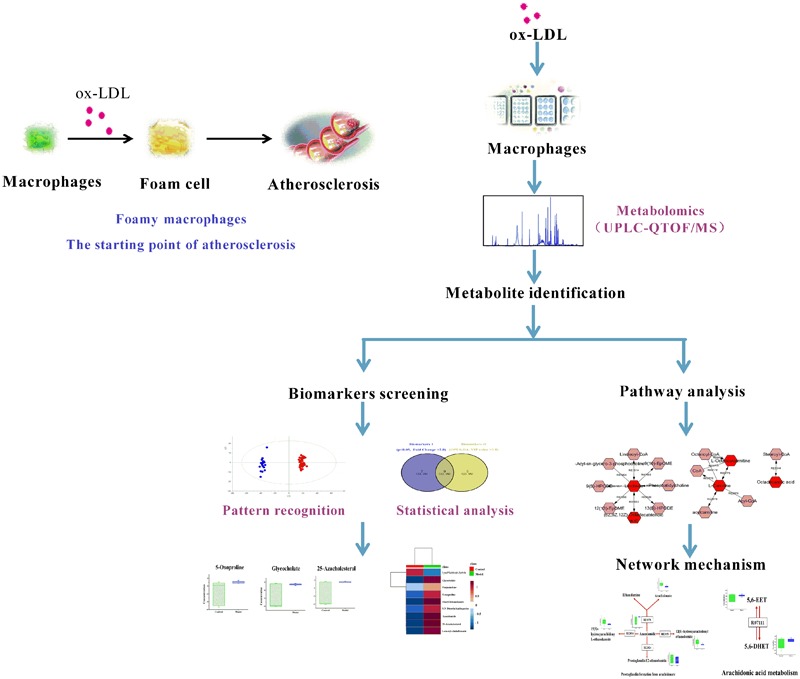
Scheme of the cell metabolomics strategy.

RAW264.7, a mouse macrophage-like cell line, was used for global metabolomics study and the detailed scheme is given in **Figure 1**.

### Ox-LDL Induced Foamy Macrophages Formation

**FIGURE 2:**
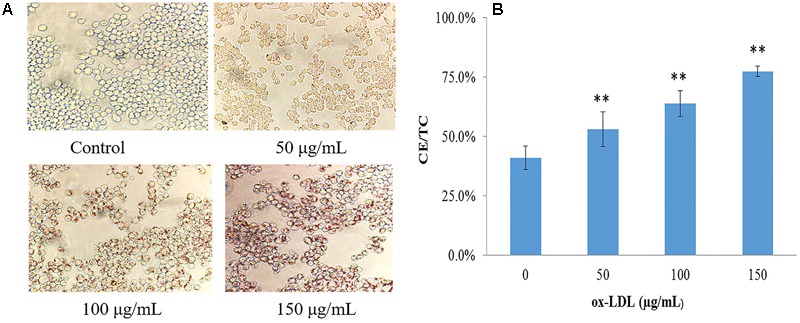
Evaluation of ox-LDL induced macrophage-derived foam cells formation. **(A)** Cell morphology after oil-red O staining (40 × 10), **(B)** intracellular lipid content of foamy macrophages, values are reported as mean ± SD (*n* = 9), ^∗∗^*p* < 0.01 vs. control.

Mouse macrophages, RAW264.7 was stimulated by ox-LDL at different concentrations to define the optimal concentration; lipid droplets staining and CE content were used as assessment indicators. Oil red O staining showed no or rare lipid droplets in normal cells; a small amount of lipid droplets can be seen in 50 μg/mL ox-LDL-induced macrophages; 100 and 150 μg/mL ox-LDL-induced lipid droplets developed in cholesterol-overloaded cells. The cell morphology stained by oil red O is shown in **Figure 2A**. When foamy macrophages formed, the intracellular FC was converted into CE, resulting in a significant rise in CE levels. The proportion of CE in normal macrophages was less than 50%, but when ox-LDL was added, the CE content increased significantly (**Figure 2B**). As 50 μg/mL of ox-LDL had milder effect on foam cell formation, and 150 μg/mL was associated with a dramatic reduction in cell viability measured by MTT, we determined that the optimal concentration of ox-LDL was 100 μg/mL.

### The Metabolic Disturbances in Ox-LDL Induced Macrophage-Derived Foam Cells

The metabolic disturbances in foamy macrophages were explored based on a global metabolomics strategy. A methyl tert-butyl ether two-phase extraction system was applied for improving lipid enrichment. The metabolite identities were confirmed based on the precise molecular mass provided by the high resolution mass spectrometry and mass spectrometry data matched against the METLIN database. METLIN represents the largest collection of mass spectrometry spectra to assist in metabolite research and identification which is an accelerated workflow for global metabolomics research (Tautenhahn et al., 2012). Ultimately, a total of 163 metabolites were identified from cell lysis based on global metabolite profiling (Supplementary Table S1). The quality of cell metabolite profile was assessed using QC samples and confirmed to meet the requirements (**Supplementary Figure S1**). Before univariate statistical analysis, metabolites with peak area error larger than 30% in QC samples were removed. In all, 30 significantly differential metabolites (*p* < 0.05) were identified between groups using Wilcoxon Mann–Whitney test.

**FIGURE 3:**
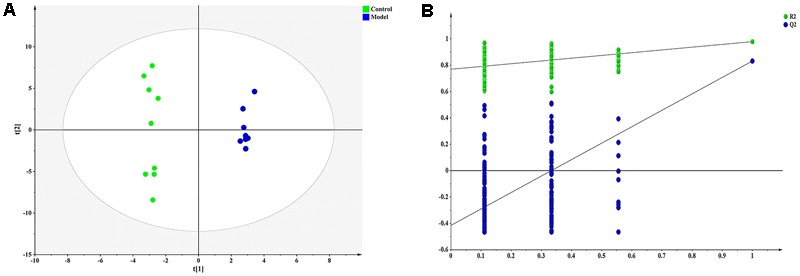
Orthogonal partial least squares discriminant analysis (OPLS-DA) result of ox-LDL treated group and control. **(A)** OPLS-DA scatter plot [*R*^2^X, 0.865; *R*^2^Y, 0.993; *Q*^2^ (cum), 0.868], **(B)** Permutation validation plots.

Pattern recognition was applied to analyze the cell metabolomics data to capture subtle metabolic perturbations in foamy macrophages. Orthogonal partial least squares discriminant analysis (OPLS-DA) was employed to establish a model for discriminating different groups. The normalized data set of the 30 characteristic metabolites (*p* < 0.05) was used as input data, with high cumulative *R*^2^ and *Q*^2^ values (**Figure 3A**). Response permutation test (200 iterations) was employed to avert the over fitting of OPLS-DA model (**Figure 3B**).

### Screening of Foamy Macrophages-Related Candidate Biomarkers

**FIGURE 4:**
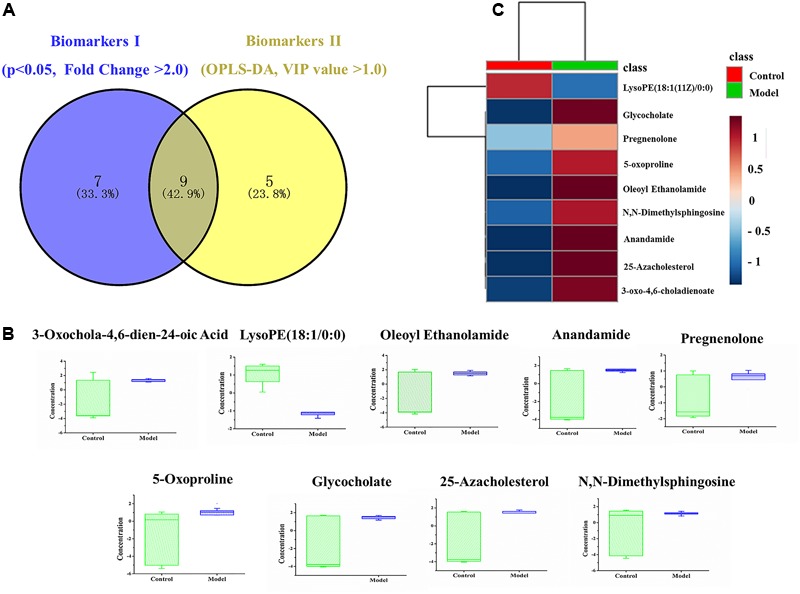
Identification and comparison of differential metabolites. **(A)** Venn diagram, **(B)** box plots of potential biomarkers, **(C)** heat-map of potential biomarkers.

To date, diagnosis of early AS still remains a great challenge. Identification of candidate metabolic biomarkers may help to measure the degree of foamy macrophages and evaluate the effect of therapeutic interventions. In this study, pattern recognition and multivariate statistical analysis were combined to refine biomarker patterns out of complex metabolomics data. Firstly, out of the 30 significantly differential metabolites, 16 compounds with *p* < 0.05 as well as fold change > 2.0 were screened as Biomarkers I (**Table 1**). Secondly, based on the VIP values of OPLS-DA model, 14 metabolites with VIP > 1.0 were identified as Biomarkers II (**Table 2**). And then, Venn diagram was constructed to refine the candidate biomarkers. Taken together, 9 metabolites with significantly altered in content, which were included in both Biomarkers I and Biomarkers II were cross-selected: 25-azacholesterol, 3-oxo-4, 6-choladienoate, anandamide, glycocholate, lysoPE (18:1/0:0), 5-oxoproline, pregnenolone, oleoyl ethanolamide and *N, N*-dimethylsphingosine. The Venn diagram and quantitative analysis of potential biomarkers are shown in **Figures 4A,B**. All of these compounds showed significant up-regulation compared with normal controls, with the exception of lysoPE (18:1/0:0). The heat-map illustrated the regulation of different biomarkers (**Figure 4C**), and the concentrations of five biomarkers (25-azacholesterol, 3-oxo-4, 6-choladienoate, anandamide, glycocholate, and oleoyl ethanolamide) displayed the most significantly up-regulated trends. As a consequence, these five metabolites were identified as biomarkers that could distinguish foamy macrophages from controls and also as predictive factors for early AS.

**Table 1:** List of candidate biomarkers screened by statistical analysis (*p* < 0.05, fold change >2.0).

Compounds	KEGG	HMDB	Lipid maps	Regulation
5,6-DHET	C14772		LMFA03050004	Up
25-Azacholesterol		HMDB01028	LMST01010212	Up
3-Oxo-4,6-Choladienoate		HMDB00476	LMST04010235	Up
5α-Androstane-3,17-dione	C00674		LMST02020085	Up
9-*cis*-Retinoic acid	C15493		LMPR01090022	Up
9,12,13-TriHOME	C14833	HMDB04708	LMFA02000014	Up
9Z-Octadecenedioic acid	C19618		LMFA01170055	Up
Anandamide	C11695		LMFA08040001	Up
γ-Nonalactone	C08501			Up
Glycocholate	C01921		LMST05030001	Up
LysoPE [18:1(11Z)/0:0]		HMDB11505		Down
*N,N*-Dimethylsphingosine	C13914	HMDB13645	LMSP01070001	Up
Oleoyl ethanolamide		HMDB02088	LMFA08040015	Up
Pregnenolone	C01953	HMDB00253	LMST02030088	Up
5-Oxoproline	C01879	HMDB00267		Up

*KEGG, Kyoto Encyclopedia of Genes and Genomes; HMDB, The Human Metabolome Database; Lipid Maps, LIPID MAPS Lipidomics Gateway.*

**Table 2:** List of candidate biomarkers screened by OPLS-DA (VIP value > 1.0).

Compounds	KEGG	HMDB	Lipid maps	Regulation
25-Azacholesterol		HMDB01028	LMST01010212	Up
3-Oxo-4,6-Choladienoate		HMDB00476	LMST04010235	Up
6β,7β-Dihydroxykaurenoic acid	C11876			Up
Amphotericin B	C06573	HMDB14819	LMPK06000002	Up
Anandamide	C11695	HMDB04080	LMFA08040001	Up
Glycocholate	C01921	HMDB00138	LMST05030001	Up
LysoPE [18:1(11Z)/0:0]		HMDB11505		Down
MG (20:0/0:0/0:0)		HMDB11572		Up
*N,N*-Dimethylsphingosine	C13914	HMDB13645	LMSP01070001	Up
Oleoyl ethanolamide		HMDB02088	LMFA08040015	Up
Pentosidine		HMDB03933		Up
PG [18:3(9Z,12Z,15Z)/18:1(9Z)]		HMDB10679		Up
Pregnenolone	C01953	HMDB00253	LMST02030088	Up
5-Oxoproline	C01879	HMDB00267		Up

*KEGG, Kyoto Encyclopedia of Genes and Genomes; HMDB, The Human Metabolome Database; Lipid Maps, LIPID MAPS Lipidomics Gateway.*

### Foamy Macrophages-Related Pathway Analysis

**FIGURE 5:**
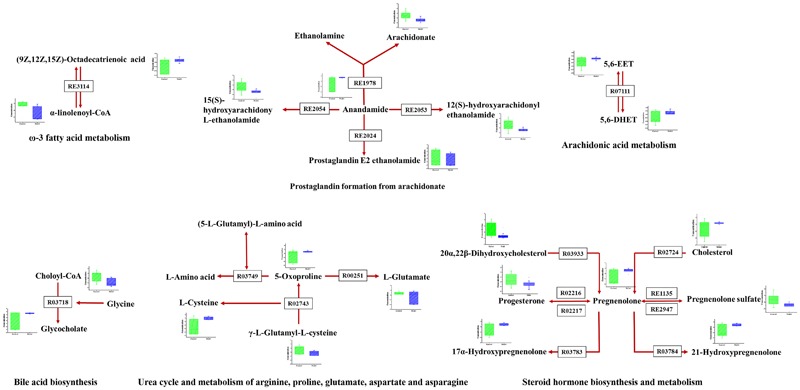
The results of pathway analysis and metabolic disturbances of related metabolites. R03718: Choloyl-CoA: glycine *N*-choloyltransferase; R03749: (5-L-Glutamyl)-L-amino-acid 5-glutamyltransferase (cyclizing); R00251:5-oxo-L-proline amidohydrolase (ATP-hydrolysing); R02743: (5-L-Glutamyl)-L-amino-acid 5-glutamyltransferase (cyclizing); R03933:20α,22β-Dihydroxycholesterol, ferredoxin: oxygen oxidoreductase (side-chain-cleaving); R02216: Pregnenolone: NAD^+^ 3-oxidoreductase; RE3114: ω-3 fatty acid metabolism; RE2054: Prostaglandin formation from arachidonate; RE1135:C21-steroid hormone biosynthesis and metabolism; R07111:5,6-EET hydrolase; R03783:pregnenolone,NADPH-hemoprotein reductase: oxygen oxidoreductase (17α-hydroxylating); R02724:Cholesterol,reduced-adrenal-ferredoxin:oxygen oxidoreductase (side-chain-cleaving); R03784: pregnenolone, NADPH-hemoprotein reductase: oxygen oxidoreductase (21-hydroxylating).

**FIGURE 6:**
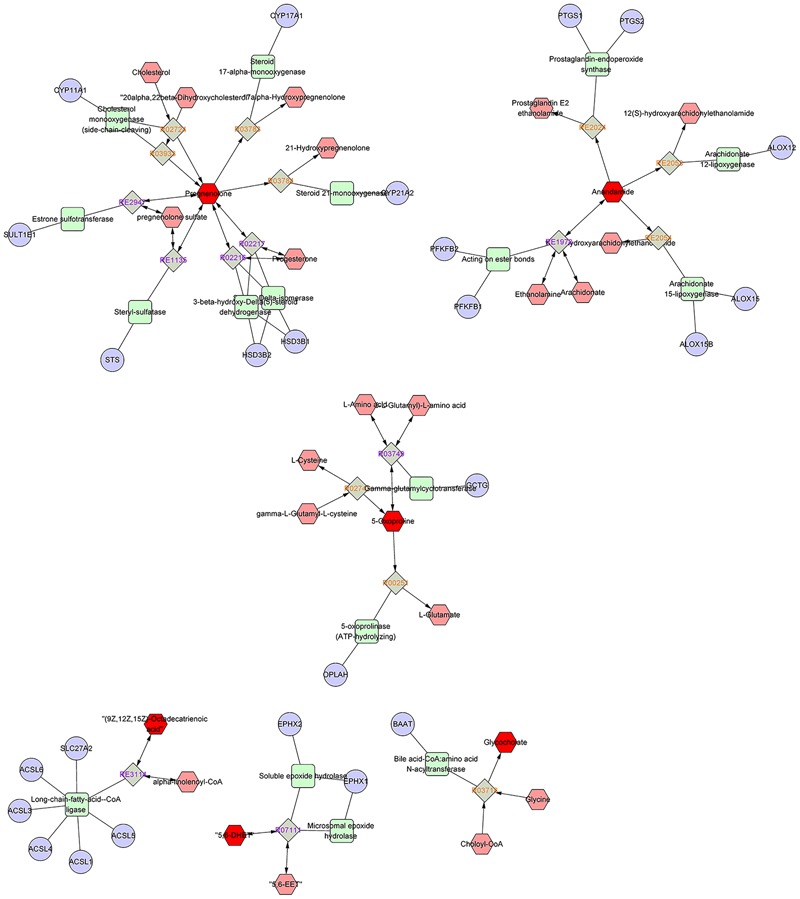
Compound-Reaction-Enzyme-Gene network map output by the metscape analysis.

As disease network could describe the complex relationship among genes, proteins and small metabolites, early AS-related metabolic network was constructed based on the relevant metabolites identified by Metscape, a plug-in of Cytoscape (**Figure 5**). Metscape is a plug-in of Cytoscape which is used for visualizing and analyzing the networks of metabolites and genes (Karnovsky et al., 2011). Significantly differential metabolites (*p* < 0.05) were imported to MetScape and recognized by name and KEGG ID, which can be used to build the Compound-Reaction-Enzyme-Gene network and the related pathways were enriched based on an internal relational database integrated with KEGG and EHMN. Pathway analysis identified six metabolic pathways related to early AS: (1) Urea cycle and metabolism of arginine, proline, glutamate, aspartate and asparagine; (2) Steroid hormone biosynthesis and metabolism; (3) Prostaglandin formation from arachidonate; (4) Bile acid biosynthesis; (5) Arachidonic acid metabolism; and (6) ω-3 fatty acid metabolism. The contents of related metabolites were also marked in the diagram, indicating that unsaturated fatty acid, especially arachidonic acid metabolism, contributed importantly to early AS (**Figure 6**). Besides, bile acid metabolism, amino acid metabolism and cholesterol metabolism also reflected significant correlation with the occurrence of AS.

## Discussion

Atherosclerosis, a multi-factorial disorder, is the underlying cause of peripheral vascular disease, coronary heart disease and cerebral infarction. Foamy macrophages as the main effector cells play a crucial role in the development and progression of AS. Therefore, it is of great significance to systematically investigate the metabolic perturbations associated with early AS based on ox-LDL-induced foamy macrophages, which may help to understand disease mechanisms and discover new targets for intervention. Cell metabolomics combined with pathway analysis is an effective platform to visualize the alteration of metabolites and unravel the pathological mechanism of complex metabolic diseases. To our knowledge, this is the first study on ox-LDL-induced foamy macrophages from the perspective of metabolomics.

### Pathological Significancy of Potential Biomarkers

Foamy macrophages formation, as a starting sign of AS, is an inflammation process in response to lipid accumulation, inspecting its metabolic disorder and filtering the corresponding biomarkers, which is beneficial to prevention or effective intervention before tissue lesions. In this study, a global view of the alterations of cell metabolites was displayed based on MTBE liquid–liquid extraction scheme, which covered different classes of metabolites (lipidome and metabolome). Five metabolites were discovered and verified as potential biomarkers of ox-LDL-induced foamy macrophages based on pattern recognition and multivariate statistical analysis. Among the five biomarkers, 25-azacholesterol could inhibit cholesterol side-chain cleavage resulting in a decrease in steroid hormone production. 3-oxo-4, 6-choladienoate, a bile acid, has been implicated in the regulation of all the key enzymes involved in cholesterol homeostasis (Chai et al., 2015). Anandamide, as the source of arachidonic acid, participates in the metabolism of unsaturated fatty acids, which is closely related to macrophage inflammation and early events of AS (Maccarrone et al., 2010). Overall, our results provide preliminary evidence for the use of these metabolites as potential biomarkers of early AS, but further large-scale clinical validations are still very essential.

### Related Pathways of Differential Metabolites

Oxidized modification lipids are considered as the major determinative factors of the occurrence and development of lesion formation (Steinberg et al., 1989). Ox-LDL was deposited in macrophages and eventually induced foam cell formation, which has been well-established to be a direct trigger and a major contributor to early AS (Moore and Tabas, 2011). Elucidate the condition of macrophages is necessary and of great practical significance to reveal typical metabolic perturbations of early AS. Metabolomics and pathway analysis of foamy macrophages indicated that there were close relationships between bile acid metabolism, amino acid metabolism and cholesterol metabolism in the occurrence of AS (**Figures 5, 6**). The increase in 5-oxoproline levels is an interesting feature, which is associated with glutamic acid metabolism and influences GSH synthesis (Shen and Sevanian, 2001). Previous studies reported that peroxide-enriched ox-LDL was a primary determining factor for GSH depletion and compensatory repletion of GSH via the up-regulated expression of γ-GCS. In our study, the substrate of GSH synthesis, γ-L-glutamyl-L-cysteine, was observed to have a down-regulated tendency based on metabolomics data, which might be associated with compensatory GSH synthesis. Metabolomics study indicated the abnormal metabolism of 5-oxoproline and GSH might be a pathological feature in ox-LDL induced macrophages. Besides, polyunsaturated fatty acid, especially arachidonic acid and its metabolites also had direct relationship with foam cell formation. Notably, arachidonic acids not only related to lipid deposition but also more importantly to pathway signaling. Lipid mediators produced by arachidonic acid are implicated in various inflammatory disorders (Levick et al., 2007). Its metabolites, including prostaglandins, leukotrienes, and hydroxyeicosatetraenoic acids, have been reported to inhibit or promote the formation of foamy macrophages (Mehrabian and Allayee, 2003; Jala and Haribabu, 2004). In our study, anandamide-related pathways are screened as a dominant pathway in the formation of foam cells. Interestingly, in anandamide-related pathways (**Figure 6**), the key metabolite (anandamide) was up-regulated, whereas the metabolic products of anandamide were down-regulated, indicating that the anandamide metabolism was hindered resulting in accumulation in foamy macrophages. Since macrophage-derived anandamide an important lipid transmitter, its decomposition in macrophages might result in a series of metabolic disorders. The metabolism obstruction of anandamide might be a typical feature of foamy macrophages, which induces the signal transduction imbalance in early-stage AS.

### Alteration in Anandamide Metabolism

Anandamide (AEA), one of the main endocannabinoids, could regulate neural regulatory mechanism or directly affect endothelial cells and vascular smooth muscle cells (Li et al., 2003). The levels of endogenous AEA are closely related to the occurrence of cardiovascular disease, and the compensatory elevation of AEA levels in tissue or plasma involved in the regulation of cardiovascular system (Matias et al., 2006). However, the compensatory mechanism of elevated AEA in cardiovascular disease and its pathophysiological significance are still unclear. Endogenous AEA elevation was observed in ox-LDL-induced macrophage-derived foam cells, which might be a compensatory change in early AS as an inflammatory response induced by ox-LDL overload.

**FIGURE 7:**
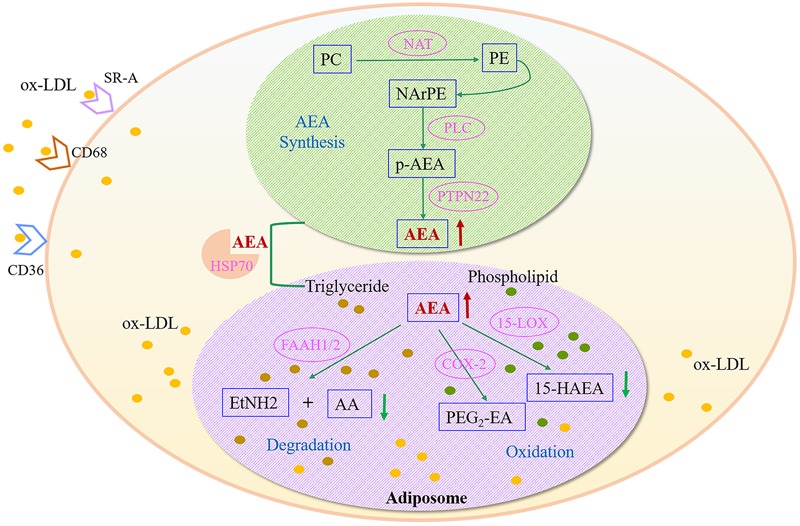
Metabolic disturbances of anandamide (AEA) in ox-LDL-induced foamy macrophages. AEA, anandamide; SR-A, Scavenger receptor-A; CD68, Macrophage a CD68; CD36, CD36 antigen; PC, phosphatidylcholine; PE, phosphatidylethanolamine; NAT, *N*-acyltransferase; NArPE, *N*-arachidonoylphosphatidylethanolamine; p-AEA, phospho-AEA; PLC, phospholipase C; HSP70, heat shock-related 70 kDa protein 2; PTPN22, protein tyrosine phosphatase non-receptor 22; FAAH, fatty acid amide hydrolase; AA, arachidonic acid; EtNH2, ethanolamine; 15-LOX, 15-lipoxygenase; 15-HAEA, 15-hydroxy-AEA; COX-2, cyclooxygenase-2; PGE2-EA, prostaglandin E2-ethanolamide.

Anandamide is found to be synthesized in neurons, endothelial cells and macrophages; unlike other neurotransmitter, it is released “on demand” instead of in storage (Okamoto et al., 2009). The biosynthetic, catabolic and oxidative pathways of AEA may work together in macrophages, and cellular uptake and intracellular transfer of AEA are critical steps of its metabolic control (Ahn et al., 2008). The possible pathways of AEA accumulation and metabolism are depicted in **Figure 7**. In macrophages, NArPE, a metabolite of phosphatidylethanolamine (PE), is converted into AEA under the action of phospholipase C and tyrosine phosphatase (PTPN22) (Liu et al., 2006). Metabolic control of AEA may be affected by ox-LDL overload, and then the release is destroyed, resulting in increased amount of AEA and its accumulation in macrophages. Ox-LDL overload leads to a rapid increase in intracellular phosphatidylethanolamine, which may contribute to abnormal AEA deposition in foamy macrophages. Once synthesized in the intracellular compartment, AEA can be trafficked to adiposomes by HSP70, where is the main occasion for AEA storage and metabolism. The major route for AEA degradation is driven by its main metabolizing enzyme fatty acid amide hydrolase (FAAH). AEA is hydrolyzed to ethanolamine and arachidonic acid, which is the key event controlling the content of endogenous AEA *in vivo*. Instead of degradation, oxidation is another route for AEA metabolism. AEA can be oxygenated by 15-lipoxygenase to 15-hydroxy-AEA (Amadio et al., 2010), or by cyclooxygenase-2 to prostaglandin E_2_-ethanolamide (Rouzer and Marnett, 2008). The two metabolic products are decreased when foamy macrophage formed (**Figure 5**), indicating that the corresponding pathway is inhibited by ox-LDL over-deposition. The direct effect of ox-LDL uptake is to increase the accumulation of intracellular lipid, mainly triglyceride and phospholipid. Lipid overload destroys the AEA synthesis under physiological conditions and induces the accumulation of intracellular AEA, resulting in interference of further signal transduction, as well as metabolic abnormalities. Thus, AEA over-accumulation might be a typical feature of ox-LDL induced foamy macrophage formation, which provides new insights into early AS from the perspective of metabolomics. Nevertheless, additional reaches are necessary for elucidating the mechanism and the physiological significance of anandamide.

### Summary

In this study, the metabolic perturbations of ox-LDL-induced macrophage-derived foam cells was investigated based on a global metabolomics platform. Foamy macrophages-specific potential biomarkers were screened by pattern recognition and multivariate statistical analysis. Five biomarkers that could effectively distinguish foamy macrophages from controls are considered as a promising tool for early diagnosis of AS. Furthermore, pathway analysis identified anandamide metabolism as a dominative pathway interrelated to foamy macrophages; the disruption of its synthesis by abnormal lipid deposition might be a potential mechanism of foamy macrophage formation. Our study offers a holistic understanding of the development and progression of early AS, and provides a reference for clinical diagnosis and discovery of new drug targets. More large-scale studies using multi-analytical techniques are still required to further validate these findings.

## Author Contributions

HX and AL designed the research. WX conducted the experiments, analyzed the data and drafted the manuscript. ZW performed the cell model and sample preparation. FD and CC helped to conduct the cell model. JD, KC, and LC co-worked on reagents, materials and prepared figures. JL and XY participated in the interpretation of the results. All authors approved the final manuscript.

## Conflict of Interest Statement

The authors declare that the research was conducted in the absence of any commercial or financial relationships that could be construed as a potential conflict of interest.

## Footnotes

1 http://www.metaboanalyst.ca/

2 http://www.cytoscape.org

## Supplementary Material

FIGURE S1 Evaluation of analytical characteristics. **(A)** QC chart time series plot for QC samples, **(B)** distributions of RSD for metabolites among all QC samples. Each column represents the percentage of compound number within corresponding RSD range. The line reveals the accumulation of percentage of responses.
