# Supplementary figures and images for: Global Metabolomics Reveals the Metabolic Dysfunction in Ox-LDL Induced Macrophage-Derived Foam Cells
Source: Front Pharmacol. 2017 Aug 31;8:586. doi: 10.3389/fphar.2017.00586 (PMC5583968; doi:10.3389/fphar.2017.00586)

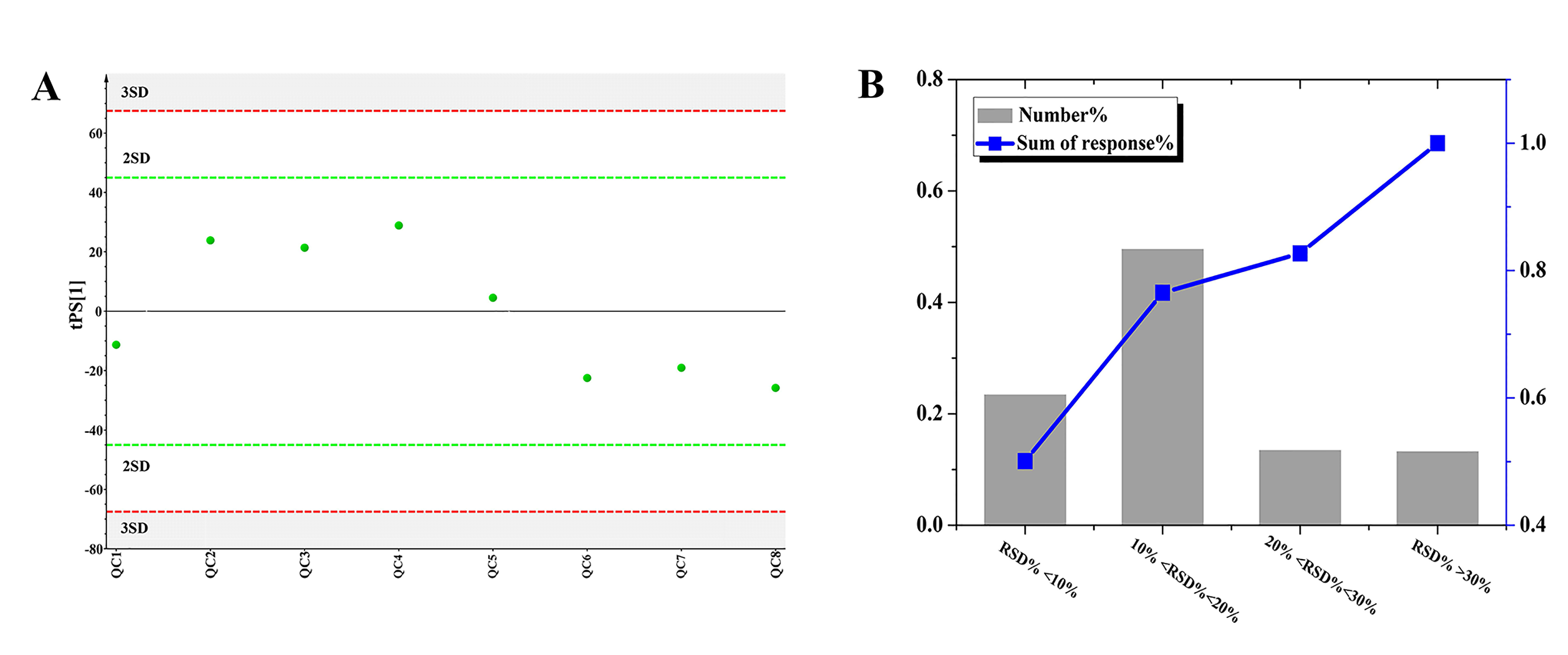

Supplement: FIGURE S1 — Evaluation of analytical characteristics. (A) QC chart time series plot for QC samples, (B) distributions of RSD for metabolites among all QC samples. Each column represents the percentage of compound number within corresponding RSD range. The line reveals the accumulation of percentage of responses. [file Image_1.TIF]
