# Supplementary material for: Global Metabolomics Reveals the Metabolic Dysfunction in Ox-LDL Induced Macrophage-Derived Foam Cells
Source: Front Pharmacol. 2017 Aug 31;8:586. doi: 10.3389/fphar.2017.00586 (PMC5583968; doi:10.3389/fphar.2017.00586)
Supplement: Supplementary file 2 [file Table_1.DOCX]

**TABLE S1| Cell metabolites obtained from ox-LDL-treated model and control groups.**

| **Compounds** | **Mass** | **Formula** | **KEGG** | **HMDB** | **Lipid Maps** | **p** | **FC** | **Regulation** |
| --- | --- | --- | --- | --- | --- | --- | --- | --- |
| 12-Methyl myristic acid | 242.2246 | C15H30O2 | C16665 |  | LMFA01020008 | 0.18 | 1.30 | up |
| Arachidonoyl ethanolamide* | 347.2824 | C22H37NO2 | C11695 | HMDB04080 | LMFA08040001 | 0.01 | 243 | up |
| 5,6-DHET* | 338.2457 | C20H34O4 | C14772 |  | LMFA03050004 | 0.03 | 2.38 | up |
| C25-Allenic-apo-aldehyde | 382.2508 | C25H34O3 | C14044 |  | LMPR01070293 | 0.04 | 43.0 | up |
| (E)-3-Hexadecenoic acid | 254.2246 | C16H30O2 |  | HMDB33791 |  | 0.06 | 1.44 | up |
| (R)-3-Hydroxy-5-phenylpentanoic acid | 194.0943 | C11H14O3 |  | HMDB31517 |  | 0.30 | 3.40 | down |
| (Z,Z)-3,6-Dodecadien-1-ol | 182.1671 | C12H22O |  | HMDB31102 |  | 0.08 | 1.48 | up |
| 4-Methoxybenzyl isothiocyanate* | 179.0405 | C_9_H_9_NOS |  | HMDB32581 |  | 0.03 | 1.72 | up |
| 10,20-Dihydroxyeicosanoic acid | 344.2927 | C20H40O4 |  | HMDB31923 |  | 0.17 | 1.30 | up |
| 10-hydroxy capric acid | 188.1412 | C10H20O3 | C02774 |  | LMFA01050033 | 0.15 | 1.37 | up |
| 10-Hydroxymyristic acid methyl ester | 258.2195 | C15H30O3 |  | HMDB31061 |  | 0.09 | 1.43 | up |
| 11-hydroxyandrosterone | 306.2195 | C19H30O3 | C14606 | HMDB02984 | LMST02020086 | 0.08 | 1.46 | up |
| 12-oxo-10E-dodecenoic acid | 212.1412 | C12H20O3 | C16309 |  | LMFA01060093 | 0.60 | 1.15 | up |
| 16,17-didehydroprogesterone | 312.2089 | C21H28O2 | C03207 |  | LMST02030163 | 0.37 | 1.48 | down |
| 16-Oxo-palmitate | 270.2195 | C16H30O3 | C19614 |  |  | 0.74 | 1.08 | down |
| 2,4,14-Eicosatrienoic acid isobutylamide | 361.3345 | C24H43NO |  | HMDB32032 |  | 0.81 | 1.02 | down |
| 2,5-Dihydro-2,4,5-trimethyloxazole | 113.1841 | C6H11NO |  | HMDB31199 |  | 0.64 | 1.11 | up |
| 25-azacholesterol* | 387.3501 | C26H45NO |  | HMDB01028 | LMST01010212 | 0.01 | 231 | up |
| 25-Cinnamoyl-vulgaroside | 566.3244 | C34H46O7 |  | HMDB41367 |  | 0.36 | 1.43 | up |
| 2-Dodecylbenzenesulfonic acid | 326.1216 | C18H30O3S |  | HMDB31031 |  | 0.66 | 1.27 | up |
| 2E-Eicosenoic acid | 310.2872 | C20H38O2 |  | HMDB02231 |  | 0.58 | 1.08 | down |
| 2-Mercaptobenzothiazole | 166.9863 | C7H5NS2 | C14437 | HMDB30524 |  | 0.08 | 21.7 | up |
| 2-methoxy-hexadecanoic acid | 286.2508 | C17H34O3 | C13947 |  | LMFA01080009 | 0.08 | 1.45 | up |
| 2-oxophytanic acid | 326.2821 | C20H38O3 | C02117 |  | LMPR0104010016 | 0.21 | 1.22 | up |
| 2-Pentadecanone | 226.2297 | C15H30O |  | HMDB31081 |  | 0.41 | 1.25 | up |
| 2Z-1-hexadecenal | 238.2297 | C16H30O | C06123 |  | LMFA06000089 | 0.24 | 1.26 | up |
| Isohexenyl-glutaconyl-CoA | 961.2095 | C32H50N7O19P3S | C01291 |  |  | 0.80 | 1.08 | up |
| 3-Acetyl-5α-androst-2-en-17β-ol* | 316.2402 | C21H32O2 | C15156 |  |  | 0.04 | 1.92 | up |
| 3β-Acetoxy-12-oxo-28,13β-oleananolide | 512.3502 | C32H48O5 |  | HMDB34646 |  | 0.11 | 33.5 | up |
| 3-ketosphinganine | 299.2824 | C18H37NO2 | C02934 | HMDB01480 | LMSP01020002 | 0.26 | 1.38 | down |
| 3-Oxochola-4,6-dien-24-oic acid* | 370.2508 | C24H34O3 |  | HMDB00476 | LMST04010235 | 0.01 | 150 | up |
| 3-oxo-dodecanoic acid | 214.1569 | C12H22O3 | C02367 | HMDB10727 | LMFA01060091 | 0.94 | 1.02 | down |
| 3-oxo-tetradecanoic acid | 242.1882 | C14H26O3 |  | HMDB10730 | LMFA01060099 | 0.35 | 3.16 | up |
| 5α-androstane-3,17-dione* | 288.2089 | C19H28O2 | C00674 |  | LMST02020085 | 0.02 | 2.10 | up |
| 5-Aminopentanoic acid | 117.0790 | C5H11NO2 | C00431 | HMDB03355 | LMFA01100040 | 0.10 | 1.35 | up |
| 5-Androstene-3b,16a,17a-triol* | 306.2195 | C19H30O3 |  | HMDB00540 | LMST02020097 | 0.02 | 2.18 | up |
| 5β-androstane-3,17-dione | 288.2089 | C19H28O2 | C03772 | HMDB03769 | LMST02020058 | 0.13 | 6.80 | up |
| 5β-Cholestane-3α,7α,12α,26-tetrol | 436.3553 | C27H48O4 | C05446 | HMDB01231 | LMST04030014 | 0.90 | 1.03 | up |
| 6β,7β-Dihydroxykaurenoic acid* | 334.2144 | C20H30O4 | C11876 |  |  | 0.02 | 1.60 | up |
| 7S,8S-DiHODE* | 312.2301 | C18H32O4 | C07354 |  | LMFA02000224 | 0.00 | 1.85 | up |
| 8-Heptadecenal | 252.2453 | C17H32O |  | HMDB41335 |  | 0.07 | 3.37 | up |
| 9,10-diHOME-(d4) | 318.2708 | C18H30D4O4 | C14828 | HMDB04704 | LMFA02000235 | 0.23 | 1.24 | up |
| 9-cis-Retinoic acid* | 300.2089 | C20H28O2 | C15493 | HMDB02369 | LMPR01090022 | 0.01 | 2.24 | up |
| 9,12,13-TriHOME* | 330.2406 | C18H34O5 | C14833 | HMDB04708 | LMFA02000014 | 0.04 | 2.19 | up |
| 9Z-Octadecenedioic acid* | 312.2301 | C18H32O4 | C19618 |  | LMFA01170055 | 0.01 | 2.05 | up |
| Actinonin | 385.2577 | C19H35N3O5 | C12056 |  |  | 0.34 | 1.83 | up |
| Aflatoxin B1 | 312.0634 | C17H12O6 | C06800 | HMDB06552 | LMPK10000006 | 0.14 | 10.8 | up |
| All trans decaprenyl diphosphate | 864.6162 | C50H90O7P2 |  | HMDB06288 |  | 0.99 | 1.00 | up |
| α-Methyl-m-tyrosine | 195.0895 | C10H13NO3 | C11820 |  |  | 0.30 | 1.11 | up |
| Aminopentol | 405.3454 | C22H47NO5 | C19805 |  |  | 0.13 | 1.33 | up |
| Cucurbic acid* | 212.1242 | C12H20O3 | C08482 |  | LMFA02020013 | 0.02 | 1.76 | up |
| Araliacerebroside | 731.5547 | C40H77NO10 |  | HMDB33621 |  | 0.07 | 1.26 | up |
| Arginyl-histidine | 311.1706 | C12H21N7O3 |  | HMDB28711 |  | 0.32 | 1.12 | down |
| Armillaramide | 555.5227 | C34H69NO4 |  | HMDB37105 |  | 0.72 | 1.16 | up |
| Avocadyne 4-acetate* | 326.2457 | C19H34O4 |  | HMDB31049 |  | 0.04 | 1.61 | up |
| Cer(d18:0/12:0) | 483.4651 | C30H61NO3 |  | HMDB11758 |  | 0.71 | 1.13 | up |
| Cer(d18:1/16:0) | 537.5121 | C34H67NO3 | C00195 | HMDB04949 | LMSP02010004 | 0.41 | 1.28 | down |
| Cholest-5-ene | 370.36 | C27H46 | C05416 | HMDB00941 | LMST01010243 | 0.36 | 1.58 | down |
| cis-Gondoic acid | 310.2872 | C20H38O2 | C16526 | HMDB02231 | LMFA01030085 | 0.69 | 1.08 | up |
| Cyclopassifloic acid E | 552.3662 | C31H52O8 |  | HMDB36298 |  | 0.49 | 1.33 | down |
| Decylubiquinol | 324.2301 | C19H32O4 | C15495 |  |  | 0.11 | 1.43 | up |
| Dehydrocarpaine I | 476.3614 | C28H48N2O4 |  | HMDB30271 |  | 0.05 | 3.69 | up |
| Dioctyl phthalate | 390.277 | C24H38O4 | C03690 |  |  | 0.09 | 2.13 | up |
| Docosatetraenoyl ethanolamide | 375.3137 | C24H41NO2 | C13829 |  | LMFA08040047 | 0.25 | 1.26 | down |
| dodecanamide | 199.1936 | C12H25NO | C13831 |  | LMFA08010001 | 0.06 | 1.48 | up |
| Eicosapentaenoic acid | 303.2246 | C20H30O2 | C06428 | HMDB01999 | LMFA01030759 | 0.95 | 1.09 | down |
| Elaidolinoleic acid* | 278.2246 | C18H30O2 | C06427 | HMDB01388 | LMFA01030153 | 0.02 | 1.65 | up |
| Fructoselysine | 308.1584 | C12H24N2O7 | C16488 |  |  | 0.05 | 41.1 | up |
| γ-Nonalactone* | 156.115 | C9H16O2 | C08501 |  |  | 0.02 | 2.11 | up |
| Ganglioside GA2 (d18:1/24:0) | 1176.8223 | C62H116N2O18 | C06135 | HMDB04897 |  | 0.10 | 1.39 | up |
| Ganglioside GM3 (d18:1/16:0) | 1152.7132 | C57H104N2O21 | C04730 | HMDB04844 |  | 0.51 | 2.36 | down |
| Gentioside | 552.1479 | C25H28O14 |  | HMDB30872 |  | 0.08 | 17.4 | up |
| Geosmin | 182.1671 | C12H22O | C16286 |  |  | 0.23 | 1.19 | up |
| Glucosylceramide (d18:1/26:1(17Z)) | 837.7058 | C50H95NO8 | C01190 | HMDB04976 | LMSP0501AA00 | 0.26 | 4.23 | down |
| Glycocholic acid* | 465.309 | C26H43NO6 | C01921 | HMDB00138 | LMST05030001 | 0.01 | 221 | up |
| Glyuranolide | 512.3138 | C31H44O6 |  | HMDB38745 |  | 0.57 | 1.83 | up |
| Heneicosanoic acid | 326.3158 | C21H42O2 |  | HMDB02345 | LMFA01010021 | 0.93 | 1.02 | down |
| Heptylmalonic acid* | 202.1205 | C10H18O4 |  | HMDB59719 |  | 0.01 | 1.73 | up |
| Hexadecanedioic acid | 286.2144 | C16H30O4 | C08260 | HMDB00672 | LMFA01170022 | 0.36 | 2.91 | down |
| Indan-1-ol | 134.0732 | C9H10O | C01710 |  |  | 0.43 | 2.50 | down |
| L-Alanyl-L-leucine | 202.1317 | C9H18N2O3 |  | HMDB28691 |  | 0.13 | 1.33 | up |
| Lauric acid | 200.1716 | C12H24O2 | C02679 | HMDB00638 | LMFA01010012 | 0.05 | 1.27 | up |
| Lauroyl diethanolamide | 287.246 | C16H33NO3 |  | HMDB32358 |  | 0.16 | 1.37 | up |
| Laurylaldehyde | 184.1827 | C12H24O | C02278 | HMDB33933 | LMFA06000071 | 0.10 | 1.33 | up |
| Leucyl-leucyl-norleucine | 357.2628 | C18H35N3O4 | C11328 |  |  | 0.23 | 1.36 | up |
| Lignoceric acid | 368.3654 | C24H48O2 | C08320 |  | LMFA01010024 | 0.25 | 1.05 | down |
| Linalyl cinnamate | 284.1766 | C19H24O2 |  | HMDB30432 |  | 0.13 | 7.37 | up |
| Linoleoyl ethanolamide | 323.2824 | C20H37NO2 |  | HMDB12252 | LMFA08040004 | 0.05 | 2.39 | up |
| L-Lactic acid | 90.0317 | C3H6O3 | C00186 |  | LMFA01050410 | 0.15 | 5.99 | up |
| L-Leucine | 131.0946 | C6H13NO2 | C00123 | HMDB00687 |  | 0.44 | 3.02 | down |
| Lucidenic acid A | 458.2668 | C27H38O6 |  | HMDB37611 |  | 0.66 | 1.41 | up |
| L-Valine | 117.079 | C5H11NO2 | C00183 | HMDB00883 |  | 0.71 | 1.02 | up |
| LysoPE(0:0/18:0) | 481.3168 | C23H48NO7P |  | HMDB11129 |  | 0.10 | 1.56 | down |
| LysoPE(15:0/0:0) | 439.2699 | C20H42NO7P |  | HMDB11502 |  | 0.13 | 6.55 | up |
| LysoPE(18:1(11Z)/0:0) * | 479.3012 | C23H46NO7P |  | HMDB11505 |  | 0.00 | 2.87 | down |
| LysoPE(24:0/0:0) | 515.4107 | C29H60NO7P |  | HMDB11527 |  | 0.32 | 1.39 | up |
| Lysyl-Tyrosine | 309.1689 | C15H23N3O4 |  | HMDB28963 |  | 0.68 | 1.29 | up |
| Melilotoside C | 898.529 | C47H78O16 |  | HMDB41466 |  | 0.26 | 4.38 | up |
| Merodesmosine | 402.2478 | C18H34N4O6 |  | HMDB30407 |  | 0.14 | 12.8 | up |
| Methyl dodecanoate | 214.1933 | C13H26O2 |  | HMDB31018 |  | 0.09 | 1.38 | up |
| Methyl N-(a-methylbutyryl)glycine* | 188.1049 | C9H16O4 | C08261 | HMDB00784 | LMFA01170054 | 0.02 | 1.78 | up |
| Methyl stearate | 298.2872 | C19H38O2 |  | HMDB34154 |  | 0.94 | 1.02 | down |
| MG(0:0/16:0/0:0) | 330.277 | C19H38O4 |  | HMDB11533 |  | 0.78 | 1.09 | up |
| MG(0:0/18:3(9Z,12Z,15Z)/0:0) | 352.2614 | C21H36O4 |  | HMDB11540 |  | 0.79 | 1.06 | up |
| MG(18:3(9Z,12Z,15Z)/0:0/0:0) * | 352.2614 | C21H36O4 |  | HMDB11570 |  | 0.01 | 1.60 | up |
| MG(20:0/0:0/0:0) * | 386.3396 | C23H46O4 |  | HMDB11572 |  | 0.02 | 1.39 | up |
| MG(P-18:0e/0:0/0:0) | 342.3134 | C21H42O3 |  | HMDB11153 |  | 0.86 | 1.04 | up |
| Myristoleic acid | 226.1933 | C14H26O2 | C08322 | HMDB02000 | LMFA01030051 | 0.15 | 1.34 | up |
| N,N-Dimethylsphingosine* | 327.3137 | C20H41NO2 | C13914 | HMDB13645 | LMSP01070001 | 0.03 | 70.9 | up |
| Nummularine B | 591.3057 | C32H41N5O6 |  | HMDB29334 |  | 0.35 | 3.55 | up |
| Oleamide | 281.2719 | C18H35NO | C19670 |  | LMFA08010004 | 0.36 | 4.34 | down |
| Oleic acid* | 282.2559 | C18H34O2 | C00712 | HMDB00207 | LMFA01030002 | 0.05 | 2.84 | up |
| Oleoyl ethanolamide* | 325.2981 | C20H39NO2 |  | HMDB02088 | LMFA08040015 | 0.01 | 287 | up |
| Oxepahyperforin | 552.3815 | C35H52O5 |  | HMDB35374 |  | 0.52 | 1.16 | up |
| Palmitic acid methyl ester | 270.2559 | C17H34O2 | C16995 |  | LMFA07010470 | 0.77 | 1.09 | up |
| Palmitic amide | 255.2562 | C16H33NO |  | HMDB12273 |  | 0.29 | 1.17 | up |
| Palmitoyl ethanolamide | 299.2824 | C18H37NO2 | C16512 | HMDB02100 | LMFA08040013 | 0.14 | 1.30 | up |
| Palmitoyl-EA | 299.2824 | C18H37NO2 |  | HMDB02100 | LMFA08040013 | 0.18 | 5.12 | up |
| PE(15:0/20:2(11Z,14Z)) | 729.5309 | C40H76NO8P | C00350 | HMDB08901 | LMGP02010000 | 0.41 | 1.11 | down |
| PE(18:3(6Z,9Z,12Z)/14:1(9Z)) | 683.4526 | C37H66NO8P | C00350 | HMDB09119 | LMGP02010000 | 0.53 | 1.05 | up |
| PE(20:2(11Z,14Z)/18:2(9Z,12Z)) | 767.5465 | C43H78NO8P | C00350 | HMDB09291 | LMGP02010000 | 0.36 | 1.04 | up |
| PE(22:0/20:4(8Z,11Z,14Z,17Z)) | 823.6091 | C47H86NO8P | C00350 | HMDB09499 | LMGP02010000 | 0.94 | 1.02 | down |
| PE(22:4(7Z,10Z,13Z,16Z)/P-18:1(9Z)) | 777.5672 | C45H80NO7P | C00350 | HMDB09612 | LMGP02010000 | 0.08 | 1.16 | up |
| PE(22:6(4Z,7Z,10Z,13Z,16Z,19Z)/20:0) | 819.5778 | C47H82NO8P | C00350 | HMDB09691 | LMGP02010000 | 0.10 | 1.14 | up |
| PE(24:1(15Z)/15:0) | 787.6091 | C44H86NO8P | C00350 | HMDB09747 | LMGP02010000 | 0.40 | 1.33 | down |
| Pentosidine* | 378.2016 | C17H26N6O4 |  | HMDB03933 |  | 0.02 | 1.59 | up |
| Petromyzonol | 394.3083 | C24H42O4 | C16258 |  | LMST04010301 | 0.90 | 1.05 | up |
| PG(16:1(9Z)/16:0) | 720.4941 | C38H73O10P |  | HMDB10585 |  | 0.50 | 1.42 | down |
| PG(18:3(9Z,12Z,15Z)/18:1(9Z)) * | 770.5098 | C42H75O10P |  | HMDB10679 |  | 0.04 | 1.23 | up |
| PGF1α | 356.2563 | C20H36O5 | C06475 | HMDB02685 | LMFA03010137 | 0.11 | 8.20 | up |
| Phytosphingosine | 317.293 | C18H39NO3 | C12144 | HMDB04610 | LMSP01030001 | 0.27 | 1.05 | up |
| PI(16:0/18:1(11Z)) | 836.5415 | C43H81O13P | C00626 | HMDB09782 |  | 0.55 | 2.21 | up |
| PI(16:2(9Z,12Z)/16:0) | 806.4945 | C41H75O13P | C00626 | HMDB09802 |  | 0.23 | 1.06 | up |
| PI(18:0/16:1(9Z)) | 836.5415 | C43H81O13P | C00626 | HMDB09806 |  | 0.14 | 2.24 | up |
| PI(18:1(11Z)/16:0) | 836.5415 | C43H81O13P | C00626 | HMDB09822 |  | 0.51 | 1.42 | down |
| p-Menthan-1-ol* | 156.1514 | C10H20O |  | HMDB37020 |  | 0.04 | 1.44 | up |
| Polyporusterone C | 156.1514 | C10H20O |  | HMDB38497 |  | 0.47 | 2.63 | up |
| Pregnenolone* | 316.2402 | C21H32O2 | C01953 | HMDB00253 | LMST02030088 | 0.03 | 3.94 | up |
| Progesterone | 314.2246 | C21H30O2 |  | HMDB01830 |  | 0.08 | 8.09 | up |
| PS(18:0/22:5(7Z,10Z,13Z,16Z,19Z)) | 837.552 | C46H80NO10P | C02737 | HMDB10166 | LMGP03010000 | 0.99 | 1.00 | up |
| Pyroglutamic acid* | 129.0426 | C5H7NO3 | C01879 | HMDB00267 | LMGP03010000 | 0.02 | 65.6 | up |
| S-(Formylmethyl)glutathione | 349.0944 | C12H19N3O7S | C14871 |  |  | 0.79 | 1.12 | up |
| Serinyl-alanine | 176.0797 | C6H12N2O4 |  | HMDB29032 |  | 0.86 | 1.02 | down |
| Sorbaldehyde | 96.0575 | C6H8O | C19249 |  | LMFA06000006 | 0.16 | 1.28 | up |
| Spermine | 202.2157 | C10H26N4 | C00750 | HMDB01256 |  | 0.61 | 1.94 | up |
| Spheroidenone | 582.4437 | C41H58O2 | C15903 |  | LMPR01070121 | 0.28 | 1.41 | up |
| Sphinganine | 301.2981 | C18H39NO2 | C00836 | HMDB00269 | LMSP01020001 | 0.68 | 1.03 | down |
| Spirolide A | 691.4448 | C42H61NO7 |  | HMDB39434 |  | 0.33 | 3.62 | down |
| Stearaldehyde | 268.2766 | C18H36O | C01838 | HMDB02384 | LMFA06000098 | 0.60 | 1.11 | down |
| Stearoylethanolamide | 327.3137 | C20H41NO2 |  | HMDB13078 | LMFA08040051 | 0.63 | 1.11 | up |
| Sterol 3-β-D-glucoside | 410.2668 | C23H38O6 | C03641 |  |  | 0.41 | 2.55 | down |
| Testosterone | 288.2089 | C19H28O2 | C00535 | HMDB00234 | LMST02020002 | 0.06 | 23.7 | up |
| Tetrahydrocurcumin | 372.1573 | C21H24O6 |  | HMDB05789 |  | 0.94 | 1.02 | up |
| trans-9-Palmitoleic acid | 254.2246 | C16H30O2 | C08362 | HMDB03229 | LMFA01030057 | 0.77 | 1.07 | up |
| trans-Dehydroandrosterone | 288.2089 | C19H28O2 | C01227 | HMDB00077 | LMST02020021 | 0.33 | 1.51 | up |
| Triethyl phosphate | 126.0082 | C2H7O4P |  | HMDB12228 |  | 0.85 | 1.03 | down |
| Triterpenoid | 552.3121 | C30H48O7S |  | HMDB04309 |  | 0.74 | 1.12 | up |
| Trolamine | 149.1052 | C6H15NO3 | C06771 |  |  | 0.27 | 3.62 | up |
| Tyromycic acid | 452.329 | C30H44O3 |  | HMDB35888 |  | 0.22 | 1.46 | up |
| Vaccenic acid | 282.2559 | C18H34O2 | C08367 | HMDB03231 | LMFA01030077 | 0.42 | 1.23 | down |
| Vanilpyruvic acid | 210.0528 | C10H10O5 |  | HMDB11714 |  | 0.07 | 1.32 | up |
| α-Linolenic acid | 278.2246 | C18H30O2 | C06427 | HMDB01388 | LMFA01030152 | 0.07 | 19.1 | up |
| α-Tocotrienol | 424.3341 | C29H44O2 | C14153 |  | LMPR02020054 | 0.40 | 1.41 | up |

* means a statistically significant difference at p < 0.0

KEGG: Kyoto Encyclopedia of Genes and Genomes; HMDB: The Human Metabolome Database; Lipid Maps: LIPID MAPS Lipidomics Gateway
